# Implications of COVID-19 for the management of chronic non-communicable diseases in sub-Saharan Africa: application of the chronic care model

**DOI:** 10.11604/pamj.supp.2020.35.24047

**Published:** 2020-06-29

**Authors:** Hubert Amu, Robert Kokou Dowou, Laud Ampomah Boateng, Elvis Enowbeyang Tarkang

**Affiliations:** 1 Department of Population and Behavioural Sciences, School of Public Health, University of Health and Allied Science, Hohoe, Ghana; 2 Department of Epidemiology and Biostatistics, School of Public Health, University of Health and Allied Science, Hohoe, Ghana; 3 Volta Regional Health Directorate, Ghana Health Service, Ho, Ghana; 4 HIV/AIDS Prevention Research Network Cameroon, Kumba, Cameroon

**Keywords:** Chronic non-communicable diseases, chronic care model, COVID-19, sub-Saharan Africa, sustainable development goal

## Abstract

About 41 million people die of chronic non-communicable diseases (CNCDs) each year, accounting for 71% of all global deaths. The high prevalence of CNCDs is particularly problematic for sub-Saharan Africa (SSA) since CNCDs are already a major cause of mortality in the sub-region. While the case fatality rate of COVID-19 is quite low, it is worth noting that people with underlying CNCDs constitute the majority of those who die from this virus. Underpinned by the chronic care model (CCM), we present a commentary on the implications of COVID-19 for the management of CNCDs in SSA. We realized that despite the World Health Organisation’s guidelines for countries to maintain essential services while putting necessary measures in place to prevent and control the spread of COVID-19, myriad of health systems and community-level factors militate against effective management of the CNCDs in SSA. This results in disruptions in management of the conditions as well as possible long-term effects such as the deterioration of the health status of CNCD patients and even deaths. Without immediate interventions to salvage the status quo, SSA countries may not be able to achieve the Sustainable Development Goal 3.4 target of reducing by one-third, premature mortality from CNCDs by the year 2030. We recommend that financial constraints could be ameliorated through short- and long-term loan facilities from the International Monetary Fund and the World Bank to augment national efforts at strengthening health systems while combating COVID-19. We also recommend increased community engagement and public education by COVID-19 response teams to enhance community support for persons living with CNCDs and to reduce social stigmatization

## Commentary

Coronaviruses constitute a large family of viruses which cause ailments ranging from the common cold to more severe diseases among humans such as the Severe Acute Respiratory Syndrome (SARS) in 2002 and Middle East Respiratory Syndrome (MERS) in 2012 [1]. In December 2019, SARS-CoV-2 became the third coronavirus after SARS and MERS to cause serious morbidity and mortality among humans. It emerged as an outbreak of a respiratory illness in Wuhan, Hubei Province, China, and later became known as 2019-nCoV (COVID-19) [1]. By March 11 2020, the World Health Organization (WHO) declared COVID-19 as a global pandemic as it had spread to most countries in all regions of the world. As of June 3 2020, 216 countries, areas, and territories recorded 6,272,098 confirmed cases and 379,044 deaths [2]. In sub-Saharan Africa (SSA), 105,948 cases were recorded [2]. Chronic non-communicable diseases (CNCDs) are a spectrum of diseases including sickle cell disease, pulmonary disease, cancers, glaucoma, hypertension, stroke, heart disease, diabetes, chronic lung disease and asthma, which tend to be of long duration and result from a combination of environmental, physiological, behavioural, and genetic factors. About 41 million people die of CNCDs each year, accounting for 71% of all global deaths [3]. The high prevalence of CNCDs is particularly problematic for SSA since CNCDs are already a major cause of mortality in the sub-region. While the case fatality rate of COVID-19 is quite low (6% as of June 3 2020) [2], it is worth noting that people with underlying CNCDs constitute the majority of those who die from the virus [4]. The high prevalence of CNCDs coupled with the rapid spread of COVID-19 in SSA comes at the backdrop that countries in the sub-region have been struggling in accelerating progress towards achievement of the Sustainable Development Goal (SDG) 3.4, which seeks to reduce by one third, premature mortality from CNCDs through effective prevention and management by the year 2030 [5]. It, therefore, becomes imperative to understand the implications of the COVID-19 pandemic on the management of CNCDs in SSA.

This paper is underpinned by the chronic care model (CCM), which was developed by the MacColl Institute for Healthcare Innovation at Group Health Cooperative in 1992 [6]. The CCM is a well-established and authenticated framework that elucidates a comprehensive approach to caring for the chronically ill, that supports increased functional and clinical outcomes. The CCM posits that improved health outcomes of individuals with CNCDs are based on indispensable components of a health care system that encourage high-quality chronic disease care. The key constructs of the model are the health system and the community, which also have sub-constructs such as self-management support, decision support, and clinical information systems [6]. These elements are designed to work in collaboration to strengthen the provider-patient relationship and improve the health outcomes of people living with CNCDs. The CCM has thus been adopted to explain how the COVID-19 pandemic makes it difficult for the sub-region to effectively manage the CNCDs and the possible implications inherent in that.

**Health systems:** the health system forms the backbone of the management of CNCDs in SSA. To ensure effective management which improves the health outcomes of people living with CNCDs, the CCM admonishes a resilient health system that provides safe and high-quality care through a dedicated and motivated team of health professionals who are well-positioned to support improvement strategies and encourage open and systematic handling of errors through the provision of quality care [6]. The health systems as a key tenet of the CCM entails the organization of health care, delivery system design, decision support, and clinical information system. The management of CNCDs under the health systems tenet of the CCM includes general services provided by health professionals irrespective of the CNCDs presented by patients. They include checking of vital signs (such as blood pressure, visual process, and temperature, pulse and respiration [TPR], laboratory tests, history taking and general education on the conditions). There are also specific services provided to patients based on the CNCDs presented and the stage of the conditions upon presentation. These include medical and surgical procedures including chemotherapy, physiotherapy, dialysis, surgeries, and prescription of medications specific to the CNCDs.

The increasing prevalence and mortality from COVID-19 with its rapidly increasing demand on health systems makes it almost impossible for the health systems of SSA countries to keep providing the CNCD management services as expected [7]. This is because, health systems in SSA even before COVID-19 were already constrained by logistical, financial, and human resource challenges, which militated against the provision of optimum CNCD care. The countries, therefore, depended largely on international donor support. With the pandemic also impacting negatively on the economies of major donors such as the USA and China, financial support to SSA is no longer forthcoming and this further constrains the ability of the health systems in the sub-region to effectively manage CNCDs. Hospitals and other health facilities which are central in the management of CNCDs are becoming overburdened with the upsurge in COVID-19 cases in the sub-region. The management of CNCDs in these health facilities thus becomes ineffective and sometimes impossible because these health facilities are mainly the places where COVID-19 patients are isolated and treated. Patients with CNCDs needing surgeries and other medical interventions to improve their health status may have to wait a little longer to receive care as the focus is on those presenting with COVID-19.

Aside from the fact that the existing inadequate number of beds in health facilities are being occupied by COVID-19 patients, the majority of the already inadequate health professionals previously designated to care for patients with CNCDs are redirected to focus on COVID-19 related activities such as attending to patients in isolation/intensive care and contact tracing. Besides, laboratories, which conduct specimen testing for CNCD patients have now focused on taking specimen for COVID-19 suspected cases as well as carrying out the test at the expense of CNCDs. Patients with CNCDs, thus, have limited access to these facilities. Moreover, infrastructure initially designated as special clinics for managing CNCDs patients are now being used as holding centres where suspected COVID-19 cases are quarantined. The meagre financial resources of health facilities have also been diverted towards the management of COVID-19 in terms of purchasing personal protective equipment (PPE) such as masks and hand gloves to the detriment of CNCDs. The diversion of health system resources towards the management of COVID-19 at the expense of CNCDs comes at the backdrop of the WHO´s recommendation and operational planning guidelines, which entreat countries to maintain the status quo regarding the provision of already existing health services to mitigate the risk of health system collapse and aggravation of patients´ health. Health systems in SSA are, however, unable to maintain the status quo due to their logistical, financial, and human resource challenges.

In several SSA countries, people are encouraged to avoid presentation to hospitals unless it is absolutely necessary. Due to the existing inadequacy of health professionals and limited PPEs, the aim is to reduce the burden of CNCDs on the health systems of SSA countries as they are already overwhelmed by the COVID-19 pandemic, and to reduce the risk of contamination of patients with CNCDs attending routine visits at hospitals. In most SSA countries, patients with CNCDs themselves, for the fear of contracting virus, avoid going to the hospitals for their reviews. Individuals developing CNCDs and needing care for the first time also avoid going to the hospitals for diagnosis and subsequent management of their conditions. The implication of the myriad of health systems challenges, precipitated by the COVID-19 pandemic entail delays in seeking prompt care by CNCD patients, the disruption of CNCD management timelines, and the deterioration of the health status of persons living with CNCDs leading to needless deaths.

**Community:** community as a tenet of the CCM entails self-management, resources mobilization and advocacy for policies that improve CNCD care [6]. Self-management support involves supports provided by health professionals and caregivers (family, friends, and neighbours) through goal setting, action planning, coping, and follow-up and preparing CNCD patients to socially and financially manage their conditions when they are in their respective communities (at home) [6]. Self-management of CNCDs by patients involves the management processes such as self-restriction (including diet restrictions), exercise, personal first aid, and use of anthropometric equipment to monitor health status which patients adopt in managing their conditions at home. The CCM recognizes caregiver roles such as emotional support, staying with the patient, helping patients with exercises, and reminding them of their self-restrictions as central in the self-management of CNCDs by patients. However, physical/social distancing, stay-at-home guidelines and restriction on people´s movement (lockdown) in the communities as part of the preventive guideline by WHO to control the spread of COVID-19, make it difficult for people living with CNCDs to receive the necessary support from family (especially the extended family), friends, and neighbours in self-managing their conditions. According to the CCM, community support is an essential element in the management CNCDs [6]. However, the rising community stigmatization of COVID-19 patients in SSA countries militates against this essential element. Thus, as the majority of people who die of COVID-19 are those with underlying CNCDs, community members erroneously tend to stigmatize people with known CNCDs whether or not they have the disease [8]. The implication is that not only would the community members be unwilling to support CNCD patients, those with CNCDs will also be reluctant to go out for check-ups.

The CCM also identifies coping strategies by patients with CNCDs as a major factor that helps in the management of the conditions [6]. Coping with CNCDs involves both emotional and activity-based practices [9]. There are different coping methods used by people with CNCDs in dealing with pain, to regulate emotions associated with CNCDs. In SSA, religion plays a major role in coping with these CNCDs [10]. As a result, many people with CNCDs rely mainly on their religious faith and leaders (such as pastors and Imams) to manage their conditions. Others depend on community-based activities such as social visits and engaging in sporting activities to cope with their conditions. However, social restrictions including the closure/suspension of religious places of worship (chapel and mosque) and social events including sporting activities by governments of the respective SSA countries make it difficult for CNCD patients to confer with their religious leaders, to pray, or to engage in social activities as strategies of coping with their conditions. The implications of the community level constraints are disruptions in self-management procedures, reduced support from caregivers, community members, and religious leaders in managing the condition which would eventually lead to the exacerbation of their conditions.

## Conclusion

Despite the WHO guidelines to all countries across the world to maintain essential services including those related to the management of CNCDs while putting necessary measures in place to prevent and control the spread of COVID-19, myriad of health systems and community-level factors militate against effective management of the CNCDs in SSA resulting in the disruptions in management of the conditions as well as possible long-term effects such as the deterioration of the health status of CNCD patients and even deaths. Without immediate interventions to salvage the status quo, SSA countries may not be able to achieve the SDG 3.4 target of reducing by one-third, premature mortality from CNCDs by the year 2030.

**Recommendations:** to mitigate the immediate and long-term irreversible negative effects of COVID-19 on the management of CNCDs in SSA, the following recommendations have been proffered: 1) Financial constraints could be ameliorated through short- and long-term loan facilities from the International Monetary Fund and the World Bank to augment national efforts at strengthening health systems in SSA to enable them to continue being resilient to manage CNCDs even as they strive to end the COVID-19 pandemic. 2) More health professionals should be employed and deployed to continue providing care for other patients including those with CNCDs while the rest focus on effectively responding to COVID-19. 3) In countries where the recruitment and deployment of more health professionals becomes challenging due to the existing financial constraints, alternative measures for the follow-up of patients with CNCDs could be implemented. These include the use of telemedicine to check-up on patients´ progress and self-management practices. Also, courier services and drones could be used to deliver CNCD medications and urgently required documents where necessary. 4) Health professionals should be adequately provided with PPEs to improve their protection against COVID-19 infections. When this is done, they would encourage patients with CNCDs to come for reviews knowing they have minimal risk of exposure to the infection. 5) Health infrastructures in SSA should be expanded and equipped with donor support to accommodate Covid-19 patients and at the same time CNCDs patients. 6) There should be increased community engagement through their leadership and public education by COVID-19 response teams of the various SSA countries to enhance community support for persons living with CNCDs and to reduce social stigmatization.

## Competing interests

The authors declare no competing interests.
